# Robotic pancreatic surgery is no substitute for experience and clinical judgment: an initial experience and literature review

**DOI:** 10.1186/1477-7819-11-160

**Published:** 2013-07-18

**Authors:** Michael Wayne, Justin Steele, Mazen Iskandar, Avram Cooperman

**Affiliations:** 1 The Pancreas and Biliary Center, New York, USA

## Abstract

Robotic pancreatic surgery offers technical advantages, and has been applied across many surgical specialties. We report an initial experience of 12 distal pancreatic resections for benign tumors from an established pancreatic center with previous general and biliary laparoscopic experience. Of a total of 12 patients, 7 were women; the mean age was 55.5 years, and the lesions included 8 distal intraductal papillary mucinous tumors, 1 insulinoma and in 3 a non-functioning neuroendocrine tumor. All operations were performed in between 90 and 180 minutes, and blood loss and hospital stay were minimal.

## Review

### Introduction

The application and popularity of robotic surgery increase annually [1]. Robotics offer several advantages including three-dimensional visualization, enhanced dexterity through articulated instruments, higher magnification of the surgery site, and ‘arms’ that provide fixed traction and exposure [1-3]. Its adaptation in pancreatic surgery has lagged for technical, philosophical and economic reasons. We are an established hepatobiliary and pancreatic center, experienced in open pancreatic, and open and laparoscopic biliary surgery, but not robotic or laparoscopic pancreatic surgery. We report our first 12 robotic pancreatic resections, and review the relevant literature.

## Methods

Between 14 November 2011 and 9 August 2012, 12 patients underwent pancreatic robotic surgery, using the DaVinci Surgical System (Intuitive Surgical Inc, 1266 Kifer Rd, Sunnyvale Calif 94066). The patients were seven women and five men. Ages ranged from 33 to 78 years, with a mean of 55.5 years and a median of 57 years. The indications for surgery were a distal intraductal papillary mucinous neoplasm (IPMN) in eight patients, an insulinoma in one patient and non-functioning neuroendocrine tumor (NET) in three patients (Table 1). The operations included one central pancreatectomy (CP), ten distal pancreatectomies without splenectomy (DP), and one distal pancreatectomy with splenectomy (DP + S).

**Table 1:** **Patient characteristics in present series (n** = **12)**

**Category**	**No.**
Gender, female:male	7:5
Age in years, mean (range)	55.5 (33 to 78)
Indications:	
Intraductal papillary mucinous neoplasm	8
Neuroendocrine tumor	3
Insulinoma	1
Operation performed:	
Distal pancreatectomy	10
Distal pancreatectomy with splenectomy	1
Central pancreatectomy	1

## Results

Operative times ranged from 1 hour and 30 minutes to 3 hours with a median of 2 hours and 50 minutes and a mean of 2 hours and 22 minutes. Blood loss was 100 cm^3^ or less in ten patients, 150 ml in one patient and 350 ml in another. The length of stay was 3 days in two patients, 4 days in nine patients, and 5 days in one patient. There were no fistulas or significant complications (Table 2).

**Table 2:** Results for present series

**Category**	**Finding**
Gender, female:male	7:5
Operative time in hours (h) and minutes (m), mean (range)	2 h and 22 m (1 h and 30 m to 3 h)
Estimated blood loss:	
<100 ml	10
100 to 200 ml	1
300 to 350 ml	1
Length of stay:	
3 days	2
4 days	9
5 days	1
Complications:	
Mortality	0
Pancreatic fistula	0
Others	0

## Discussion

In 2003, Giulianotti *et al*. reported a series of robotic abdominal operations including the first pancreatic resection [4], and Melvin *et al*. reported a robotic excision of a neuroendocrine tumor [5]. While robotic operations have tripled between 2007 and 2010, there have been few reports of robotic pancreatic surgery [1,6]. Until recently most pancreatic surgery was performed for pancreatic adenocarcinoma, and most often required a Whipple operation, which can be intricate, lengthy and with significant morbidity [7,8]. Most distal pancreatic adenocarcinomas are large, rarely resectable or curable [7]. Why add robotics, time, cost and difficulty to an already grim situation?

The worldwide increase in body imaging has detected smaller, asymptomatic, and incidental pancreatic lesions [9,10]. These include cystic and neuroendocrine tumors, which have a far better prognosis than adenocarcinoma [9,10]. When surgery is indicated, the small size and absent vascular involvement favor laparoscopy or robotics. Most robotic pancreatic procedures (60% to 70%) are performed for benign lesions less than 3 cm, reflecting selection bias and sound judgment [2,3,11,12].

We were late to adopt robotic pancreatic surgery since open distal resections took less than 1 h and pancreaticoduodenal resections 2.5 h, both performed with minimal blood loss (<150 cm^3^), short hospital stay (5 to 8 days) and few pancreatic fistulas (0% to 6%) [13,14]. We reconsidered, as patients inquired and requested robotics. Our first robotic resection took 3 hours, and the most recent 1 hour and 30 minutes. The mean time was 2 hours and 22 minutes. There are four published case series of robotic distal pancreatectomy. The number of patients and operative times for each were: 17 patients (4 hours and 58 minutes) [2], 20 patients (5 hours and 40 minutes) [3], 30 patients (4 hours and 5 minutes) [11] and 46 patients (5 hours and 31 minutes) [12] (Table 3).

**Table 3:** Reported outcomes of robotic distal pancreatectomies

**Reference**	**Patients, n**	**Operative time, hours (h) and minutes (m)**	**Length of stay, days**	**Blood loss, ml**	**Fistulas**
Waters *et al*. [2]	17	4 h and 58 m	4	279	0
Kang *et al.*[3]	20	4 h and 58 m	7.18	372	-
Daoudi *et al.*[11]	30	4 h and 53 m	6.1	212	14
Giulianotti *et al.*[12]	46	5 h and 31 m	9.3	323	9

Our series included one central resection. Three reports of robotic central resection include three, five, and nine patients with mean operating times from 5 hours and 20 minutes to 8 hours 0” [1,14,15]. The fistula rates were one in three [15], one in five [16] and seven in nine [1] and the hospital stay ranged from 9 to 28 days. The operating time was influenced by the distal pancreatic anastomosis, that is, pancreaticogastrostomy, or pancreaticojejunostomy. We favor closure rather than anastomoses of the distal duct in central resections, as it shortens hospital stay, and operating time, minimizes fistula rates and does not increase exocrine insufficiency [17].

The robotic pancreatic studies indicate satisfaction with robotics, fewer conversions to open surgery and greater splenic preservation as compared to laparoscopy [1,3]. A large laparoscopic pancreatic experience generally precedes pancreatic robotic surgery. Despite this experience, most reported robotic operations took at least 5 h, double our initial experience. Our lack of laparoscopic pancreatic experience was balanced by the benefits of a large open experience, which provided intimate familiarity with pancreatic anatomy, and insights into case selection, and intraoperative decision making.

The cost of robotic surgery is a valid concern. Waters *et al*. compared the cost of robotic distal pancreatectomy (R) to open (O) and laparoscopic distal pancreatectomy (L) in 77 cases [2]. The mean operating times and hospital stay were for O (3 hours and 42 minutes; 8 days), for L (4 hours and 5 minutes; 6 days), and for R (4 hours and 58 minutes; 4 days). The total cost for R, O and L were US$10,588, US$12,986, and US$16,059. The higher operative cost for robotics was offset by a shorter stay for robotic procedures.

Our operating room charges were slightly higher for robotics (R) compared to open surgery (O) US$2,180.48 versus US$1,750.09 but were offset by a shorter hospital stay, 3.9 days (R) versus 6.5 (O). Kang *et al*. noted in Korea that costs were 2.5 times higher for robotic versus laparoscopic distal pancreatic resections, not offset by hospital costs. [3].

Robotic distal pancreatectomy is safe, and its inherent advantages benefit patients and facilitate surgery in appropriately selected cases.

## Conclusions

In all, 12 patients with benign pancreatic tumors underwent robotic pancreatic distal resection (11), and central resection (1). Operative times ranged from 1 hour and 30 minutes to 3 hours, blood loss less than 100 cm^3^ in ten patients, 150 cm^3^ in one patient and 350 cm^3^ in another. Hospital stay was 3 to 4 days for 11 patients and 5 days for 1 patient. Careful selection of patients and a large pancreatic experience allow satisfactory outcomes after pancreatic resections and may equalize or outweigh minimally invasive experience.

## Competing interests

The authors declare that they have no competing interests.

## Authors’ contributions

MW and JS provided the clinical data, followup on each patient and edited the manuscript; MI gathered the literature, followed up with each patient and with AC wrote the manuscript. AC formated, wrote (withMI ), and edited the manuscript. All authors declare they read and approve the manuscript.
